# EcoEvoApps: Interactive apps for theoretical models in ecology and evolutionary biology

**DOI:** 10.1002/ece3.9556

**Published:** 2022-12-03

**Authors:** Rosa M. McGuire, Kenji T. Hayashi, Xinyi Yan, Marcel Caritá Vaz, Damla Cinoğlu, Madeline C. Cowen, Alejandra Martínez‐Blancas, Lauren L. Sullivan, Sheila Vazquez‐Morales, Gaurav S. Kandlikar

**Affiliations:** ^1^ Department of Ecology and Evolutionary Biology University of California Los Angeles California USA; ^2^ Department of Integrative Biology University of Texas at Austin Austin Texas USA; ^3^ Institute for Environmental Science and Sustainability Wilkes University Wilkes‐Barre Pennsylvania USA; ^4^ Departamento de Ecología y Recursos Naturales, Facultad de Ciencias Universidad Nacional Autónoma de Mexico Ciudad de México Mexico; ^5^ Division of Biological Sciences University of Missouri Columbia Missouri USA; ^6^ Department of Plant Biology Kellogg Biological Station Michigan State University East Lansing Michigan USA; ^7^ Division of Plant Sciences & Technology University of Missouri Columbia Missouri USA

**Keywords:** ecological theory, mathematical modeling, R package, shiny apps, teaching

## Abstract

The integration of theory and data drives progress in science, but a persistent barrier to such integration in ecology and evolutionary biology is that theory is often developed and expressed in the form of mathematical models that can feel daunting and inaccessible for students and empiricists with variable quantitative training and attitudes towards math. A promising way to make mathematical models more approachable is to embed them into interactive tools with which one can visually evaluate model structures and directly explore model outcomes through simulation. To promote such interactive learning of quantitative models, we developed EcoEvoApps, a collection of free, open‐source, and multilingual R/Shiny apps that include model overviews, interactive model simulations, and code to implement these models directly in R. The package currently focuses on canonical models of population dynamics, species interactions, and landscape ecology. These apps help illustrate fundamental results from theoretical ecology and can serve as valuable teaching tools in classroom settings. We present data from student surveys which show that students rate these apps as useful learning tools, and that using interactive apps leads to substantial gains in students' interest and confidence in working with mathematical models. This points to the potential for interactive activities to make theoretical models more accessible to a wider audience, and thus facilitate the feedback between theory and data across ecology and evolutionary biology.

## Graphical abstract

EcoEvoApps presents a suite of free, customizable interactive apps with which users can explore the dynamics of canonical mathematical models. We present classroom survey data that show that use of these apps can boost student engagement and learning. The apps are available in 5 languages (English, Spanish, Chinese, Portuguese, and Turkish), and the package is developed collaboratively in a way that enables new contributions from any interested members of the community.

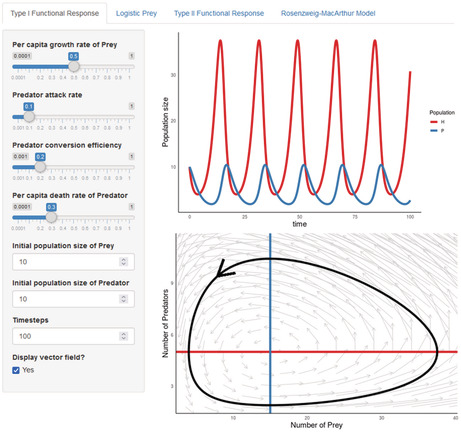

## 1 INTRODUCTION

Integrating theory with insights from observations and experiments is a fundamental driver of progress across the life sciences (Cohen, 2004; Jungck, 1997; Shou et al., 2015), including in ecology and evolutionary biology (EEB) (Marquet et al., 2014; Servedio et al., 2014). While not all theory is mathematical, research that synthesizes data with mathematical models can enable generalization across systems, promote a deeper conceptual understanding of biological systems by clarifying the role and consequences of different biological factors, reveal otherwise undetectable patterns in biological data, help disentangle complex interactions and feedbacks, and highlight important areas for further study (Caswell, 1988; Cohen, 2004; Haldane, 1964; Marquet et al., 2014; Zuk & Travisano, 2018). Such integration can also have important applications in biological forecasts and in informing actions and policies at the interface of science and society (Conway, 1977; Wainwright et al., 2018). Despite widespread agreement between empiricists and theoreticians that more synergism between these two approaches towards EEB research can yield fruitful insights (Haller, 2014; Jeltsch et al., 2013; Scheiner, 2013; Shou et al., 2015), there are numerous barriers that limit such integration.

One such barrier towards more integration is that the language of mathematical models and their analytical solutions may seem foreign to those who come to EEB from a more empirical background. As a result, equation‐heavy papers tend to be cited less often (Fawcett & Higginson, 2012), and instructors of quantitative courses tend to receive worse student evaluations than those who de‐emphasize quantitative topics (Kreitzer & Sweet‐Cushman, 2021; Uttl et al., 2013). Across quantitative biology more broadly, a growing body of research suggests that interactive tools that allow users to independently explore model structure and outcomes could help increase student interest and understanding of quantitative concepts (Feser et al., 2013; Ou et al., 2022; Thompson et al., 2010). However, while many authors have called for an increased emphasis on quantitative training at all stages in EEB education, these calls focus primarily on an increased emphasis on statistical models (e.g. Ellison & Dennis, 2010) or on programming/computational skills (e.g. Feng et al., 2020; Losos et al., 2013), with relatively few advances in the pedagogy of theoretical models (but see Grainger et al., 2022; Lehman et al., 2020). Moreover, existing pedagogical tools tend to be behind paywalls (e.g. Simbio, https://simbio.com/), written in programming languages that are not commonly used by ecologists (e.g. Populus, Alstad, 2001), and predominantly written in English. These limitations are an equity issue, and they pose multiple barriers to students and educators who wish to use and adapt such resources. Establishing an open‐source and multilingual platform for interactive simulations of EEB models thus has the dual potential to enhance equity in EEB education and to facilitate communication and collaboration between theoretical and empirical researchers.

Here we describe EcoEvoApps, an open‐source R package (ecoevoapps) and website (https://ecoevoapps.gitlab.io) that provides a collection of freely available interactive Shiny (Chang et al., 2022) apps that simulate fundamental EEB models. The package also includes functions to directly run models in R (R Core Team, 2022), and can thus serve as a bridge to help users become familiar with coding and implementing theoretical models. We illustrate how these apps can be used to help communicate and learn insights from theoretical models, both at the level of an individual seeking to gain more familiarity with a model, and in large undergraduate classroom settings. We actively invite anyone who wishes to contribute to the project by writing new apps, reviewing and/or adding new features to existing apps, translating apps into other languages, or contributing teaching plans, to join our community. Note that throughout this paper, we use "ecoevoapps" to refer to the R package specifically, and "EcoEvoApps" to refer to the project as a whole.

## 2 PACKAGE OVERVIEW

### 2.1 Interactive (shiny) apps

**FIGURE 1:**
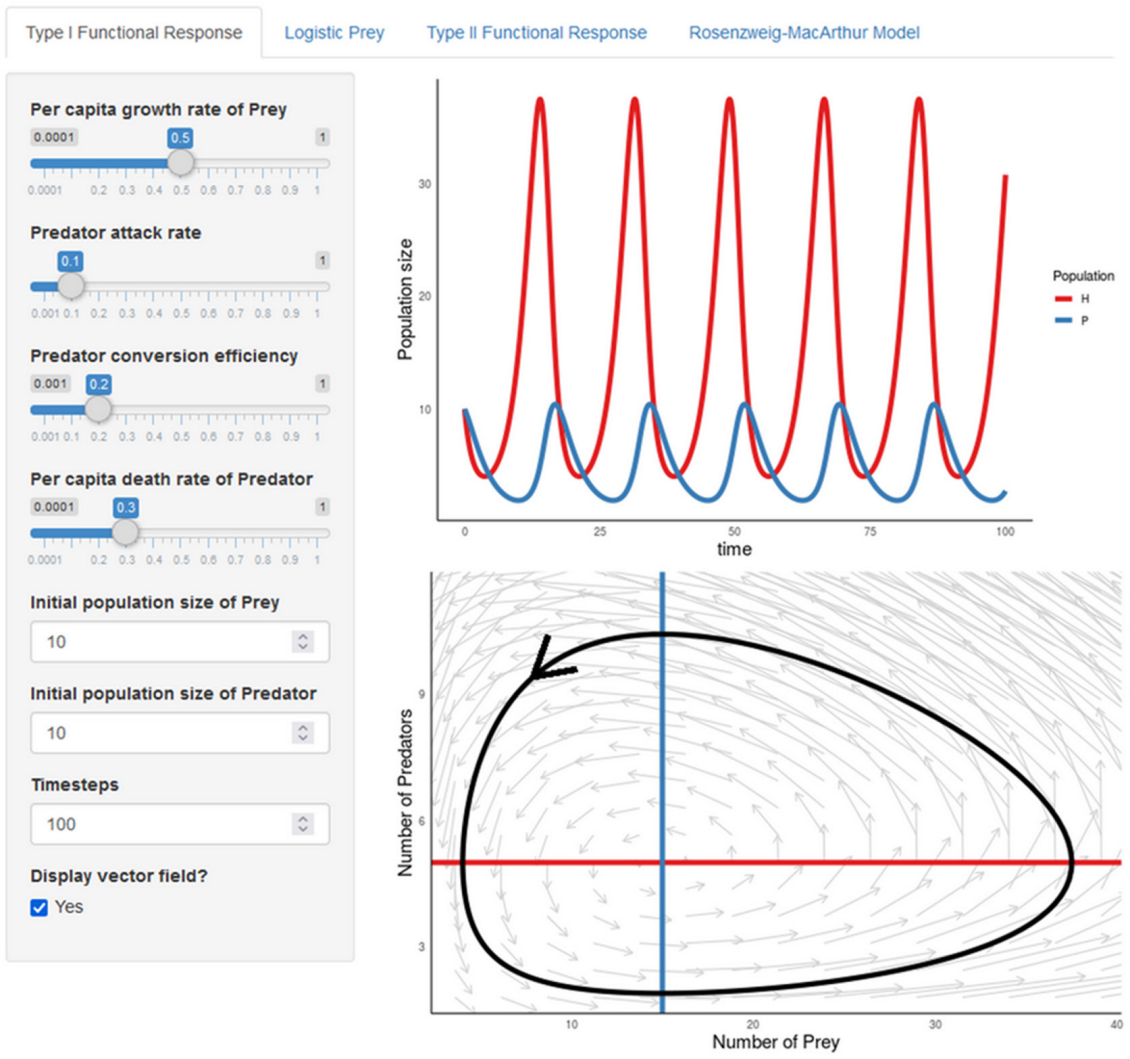
Screenshot of the predator–prey dynamics app being used to simulate Lotka‐Volterra dynamics. Users set parameter values on the left‐hand panel, and these inputs are used to generate the population trajectory and phase portrait plots on the right. In addition to this interactive component, the app also includes a verbal description of the model, the model equations, and a parameter table. Users can select three other tabs that incorporate logistic growth in the prey, type II functional response for the predator, or both.

At the heart of ecoevoapps are 12 interactive apps (Table 1), which we expect to be the primary avenue through which most users interact with the package. We chose the models to include in this first release of ecoevoapps by surveying syllabi for undergraduate ecology courses and commonly‐used textbooks (Begon & Townsend, 2020; Gotelli, 2008). We expect to build on this collection with future releases of the package. Some apps implement the dynamics of one specific model (e.g. the abiotic resource competition app, which models two species competing for two essential resources (Tilman, 1980)), while other apps present several closely related models. For example, the predator–prey dynamics app includes a tab that presents the classic Lotka‐Volterra model, and other tabs with model extensions that integrate logistic growth in the prey and/or a type II functional response for the predator (Figure 1). Each app includes a brief description of the model's background, structure, and parameters, as well as references to relevant literature. A core set of ten apps are available in English, Spanish, Chinese, Turkish, and Portuguese (Table 1). We plan to continue adding new apps and translating existing apps both internally and by soliciting contributions from community members (see “Contributing to EcoEvoApps” below).

**TABLE 1:** Models and functions included in the ecoevoapps package

Model	Link to shiny app	Functions for running the models/shiny apps	Functions for plotting model outputs
Population dynamics in continuous time	中文; Español; English; português; Turkish	run_exponential_model() run_logistic_model() shiny_singlepop_continuous()	plot_continuous_population_growth()
Population dynamics in discrete time	中文; Español; English; português; Turkish	run_discrete_exponential_model() run_discrete_logistic_model() run_beverton_holt_model() run_ricker_model() shiny_population_growth_discrete()	plot_discrete_population_growth() plot_discrete_population_cobweb()
Structured population growth	中文; Español; English; português; Turkish	run_structured_population_simulation() shiny_structured_population()	plot_leslie_diagram() plot_structured_population_size() plot_structured_population_lambda() plot_structured_population_agedist()
Mutualism	中文; Español; English; português; Turkish	run_mutualism() shiny_mutualism()	plot_mutualism_time() plot_mutualism_portrait()
Lotka‐Volterra competition	中文; Español; English; português; Turkish	run_lvcomp_model() shiny_lvcomp_model()	plot_lvcomp_time() plot_lvcomp_portrait()
Predator–prey dynamics	中文; Español; English; português; Turkish	run_predprey_model() shiny_predprey()	plot_predprey_time() plot_predprey_portrait()
Competition for abiotic resources	中文; Español; English; português; Turkish	run_abiotic_comp_model() run_abiotic_comp_rstar() shiny_abiotic_comp()	plot_abiotic_comp_time() plot_abiotic_comp_portrait()
Competition for a biotic resource	中文; Español; English; português; Turkish	run_biotic_comp_model() shiny_biotic_comp()	plot_biotic_comp_time() plot_functional_responses()
Compartment models of infectious disease dynamics	中文; Español; English; português; Turkish	run_infectiousdisease_model() shiny_infectious_disease()	plot_infectiousdisease_time() plot_infectiousdisease_portrait()
Island biogeography	中文; Español; English; português; Turkish	run_ibiogeo_model() shiny_ibiogeo_model()	none (run_ibiogeo_model() itself returns plots)
Smith‐Fretwell model (seed size/number trade‐off)	English	run_smithfretwell_model() shiny_smith_fretwell()	plot_smithfretwell_model()
Metapopulation dynamics	English	run_source_sink() shiny_source_sink()	plot_source_sink()

The Shiny apps are freely available online on RStudio's shinyapps.io servers (links available in Table 1), or can be launched locally on users' personal computers from the R console. The ecoevoapps package provides a series of functions with the prefix shiny_ that launch the apps, and a vignette with instructions for users who wish to customize and deploy their own instance of an app, e.g. for hosting on institutional servers or to modify an app's content for a specific classroom lesson. Finally, the package also includes model‐specific vignettes with instructions for simulating the model dynamics directly through R (complete list at https://ecoevoapps.gitlab.io/docs/articles/).

### 2.2 Functions for simulating and visualizing model dynamics

Under the hood, the Shiny apps use functions in the ecoevoapps package to simulate and visualize model dynamics (Table 1). Simulations are conducted by functions with the prefix run_, which take as their input the parameter values and other relevant information for the particular model. For example, run_predprey_model() requires as inputs a vector defining the parameter values (params), a vector of the initial population sizes for the predator and prey species (init), and the time steps over which to run the model (time). The function returns a data frame of the population sizes for each species over the specified time series. The package also includes a series of plotting functions prefixed plot_, which take as their input the object returned by the corresponding run_ function, and in turn return a ggplot2 object. Using ecoevoapps functions, the outputs in Figure 1 can be generated with the following code:

*# remotes::install_gitlab(“ecoevoapps/ecoevoapps”)* library(ecoevoapps) *# define parameter vector, initial state, and time* params_vec < − c(r = 0.5, a = 0.1, e = 0.2, d = 0.3) init_vec < − c(H = 10, *p* = 10) timesteps <− 100 *# Run model dynamics* lvpp_out <− run_predprey_model(params = params_vec, init = init_vec, time = timesteps) *# Plot trajectory through time* plot_predprey_time(lvpp_out) *# Plot the phase portrait* plot_predprey_portrait(lvpp_out, params_vec, vectors_field = T).

The complete list of functions for simulating and plotting model dynamics is provided in Table 1. Each function's usage is documented in the package, and suites of functions relevant to different models are described in the corresponding vignettes. While we expect the interactive apps to be the primary mode for most users' engagement with the package, users familiar with R — or those who wish to build this familiarity — can use these functions to conduct visualizations or analyses beyond those presented in the apps. Thus, the package can also serve as a gateway for users to implement and manipulate mathematical models at the command line.

### 2.3 Installation and dependencies

The ecoevoapps package can be installed from GitLab: remotes::install_gitlab(“ecoevoapps/ecoevoapps”). The package depends on functions from deSolve (Soetaert et al., 2010), diagram (Soetaert, 2020), kableExtra (Zhu, 2021), patchwork (Pedersen, 2020), rmarkdown (Allaire et al., 2022), shiny (Chang et al., 2022), and various packages within the tidyverse (Wickham et al., 2019). We have tested the ecoevoapps package on R versions >4, and have tested the Shiny apps on Firefox, Chrome, and Safari.

### 2.4 Contributing to EcoEvoApps

This manuscript describes the first release of ecoevoapps, and we envision this package to grow as a collaborative and inclusive effort. In particular, our overarching goal is to leverage the diverse expertise of the EEB community to build an open educational resource that facilitates dialogue between theoretical and empirical research. As such, we provide several mechanisms by which educators, researchers, and students can contribute to the project. These mechanisms include (1) writing and contributing new apps, (2) revising existing apps, (3) providing feedback, translating apps, or requesting new apps or features, and (4) contributing classroom activities or other use‐cases involving the use of one or more of the apps. Detailed contribution guidelines are provided as a vignette (https://ecoevoapps.gitlab.io/docs/articles/contributing.html). Contributors are acknowledged in the package source code, as well as on the project homepage (https://ecoevoapps.gitlab.io/people/).

## 3 USE CASES

### 3.1 Classroom teaching

We expect that a primary use of EcoEvoApps will be as an instructional tool to promote active learning of mathematical models in undergraduate or graduate classrooms. Instructors can incorporate these apps into lesson plans in a variety of contexts. For example, instructors who use a flipped‐classroom design (i.e. classes where students learn new information through video lectures or other media outside of class, and apply their learning through activities while in the classroom, e.g. Garcia‐Vedrenne et al., 2020; McGraw & Chandler, 2015) can pair traditional textbook reading assignments with scenarios for students to explore using an interactive app. In‐class discussions can then center on analytical solutions to the model or on students' observations of model dynamics from the app. Elements of EcoEvoApps can also feature directly in in‐class activities. Instructors of courses focusing on conceptual insights can design worksheets that use the interactive web apps to guide students through different outcomes in mathematical models, while instructors of courses that emphasize skill building could develop exercises in which students use functions in the ecoevoapps package to simulate model dynamics in R. We see developing openly available lesson plans that integrate EcoEvoApps into EEB instruction as an important next step in this project (see mechanism 4 in “Contributing to EcoEvoApps”).

As a preliminary investigation of the value of these apps in classroom settings, we conducted surveys of students who used EcoEvoApps in two undergraduate ecology courses. Classroom surveys were reviewed by the UCLA Institutional Review Board and MU Institutional Review Board and were determined to constitute “exempt” studies (UCLA IRB #20–002179; MU IRB Project #2031063, Review #276104). Detailed survey methods and questions are available in Supplement S1 and S2.

**FIGURE 2:**
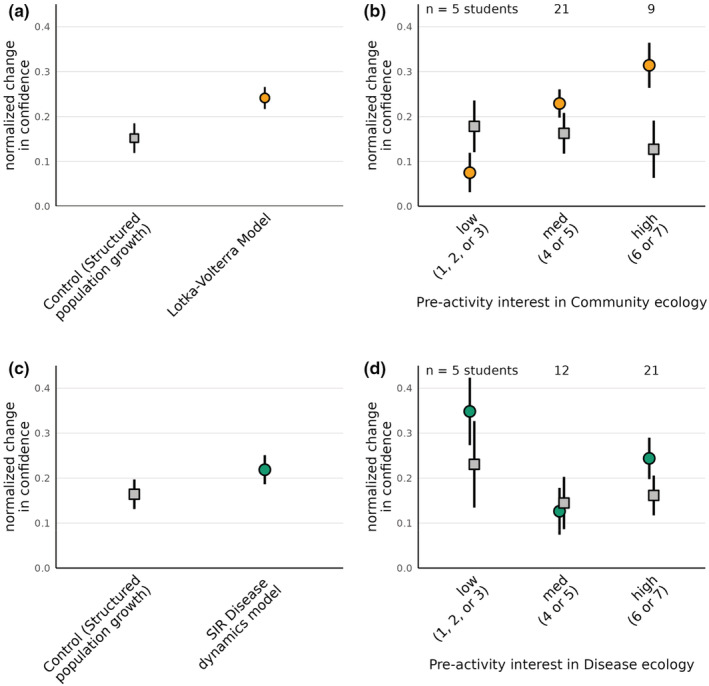
Survey responses of students who used the Lotka‐Volterra competition and SIR disease dynamics apps at the University of Missouri. Using the Lotka‐Volterra competition app led to substantial gains in student understanding (panel a), especially for students who reported high interest in community ecology prior to the activity (b). Gains in confidence were more modest from the SIR disease dynamics app (c), and largely unrelated to students' interest in disease ecology (d). Gray squares show changes in student confidence in structured population growth dynamics, which serves as a control topic. Normalized gain was calculated according to Marx and Cummings (2007); see Supplement S1 for details.

In an upper‐division General Ecology course at the University of Missouri, Columbia (MU), 37 students used the interactive apps to learn about Lotka‐Volterra competition and SIR models of infectious disease dynamics. After in‐class lectures that introduced students to the relevant modeling framework, assumptions, and equations, students completed a worksheet exercise that guided them to explore various model outputs based on different parameter inputs (Supplement S3). Before and after this worksheet activity, students completed surveys about their interest and confidence in the focal model. The worksheet also included questions about students' interest in structured population growth models, which served as a negative control. We evaluated whether using the interactive apps changed students' self reported confidence between the pre‐ and post‐activity surveys by computing the normalized change metric of Marx and Cummings (2007) (see Supplement S1 for details). Survey results indicated that using interactive apps led to substantial gains in student confidence, especially for the Lotka‐Volterra competition model (Figure 2a). Moreover, students who reported being highly interested in Community Ecology reported the highest gains in confidence from using the Lotka‐Volterra competition app (Figure 2b). Gains in confidence for the SIR model were more modest and largely unrelated to students' pre‐activity interest in disease ecology (Figure 2c,d).

**FIGURE 3:**
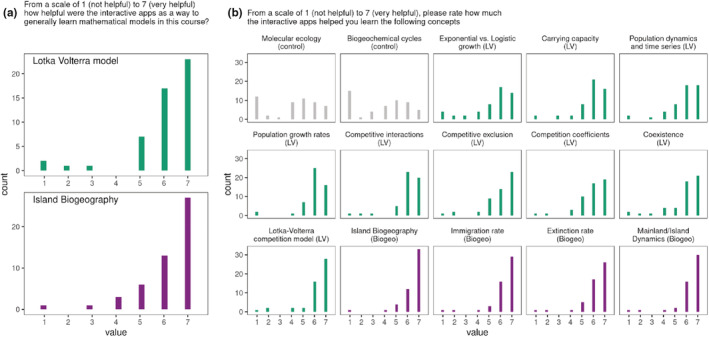
Students at UCLA (*n* = 51) generally rated the Lotka‐Volterra competition and island biogeography apps to be valuable tools to help learn the models overall (a), as well as for specific topics within each model (b). Green histograms indicate topics related to the Lotka‐Volterra competition model, purple histograms indicate topics related to island biogeography, and gray histograms indicate topics unrelated to either activity, which served as a control.

We also conducted a complementary survey of students in an upper‐division Ecology course at the University of California, Los Angeles (“UCLA”), where 51 students used the interactive apps to learn about Island Biogeography and Lotka‐Volterra competition. The learning activity included short (~15 min) lecture overviews of the model, followed by a guided exploration of the model using the relevant interactive app (Supplement S3). After the activity, students completed a survey in which they rated, on a scale of 1 (not helpful) to 7 (very helpful), the degree to which the apps helped them understand the model as a whole, as well as specific topics associated with the model. An overwhelming majority of students (40/51) reported that the apps were moderately to very helpful for learning the models (response of 6 or 7, Figure 3a). The apps also appear to help students better understand specific ideas related to the models (e.g. students report that they better understand the concepts of “carrying capacity” or “coexistence” after using the Lotka‐Volterra competition app, Figure 3b).

While these surveys hint at the value of EcoEvoApps as classroom teaching tools, more detailed pedagogical studies of whether and how interactive apps of mathematical models enhance students' understanding and interest in quantitative models in EEB is an important next step. Using validated tools like the Math‐Biology Values Instruments (Andrews et al., 2017) to evaluate the efficacy of classroom activities should be a key priority for such work, and we hope to foster a feedback loop between instructors and software developers to improve the educational value of these apps.

### 3.2 Communicating and learning insights from classic and new models

**FIGURE 4:**
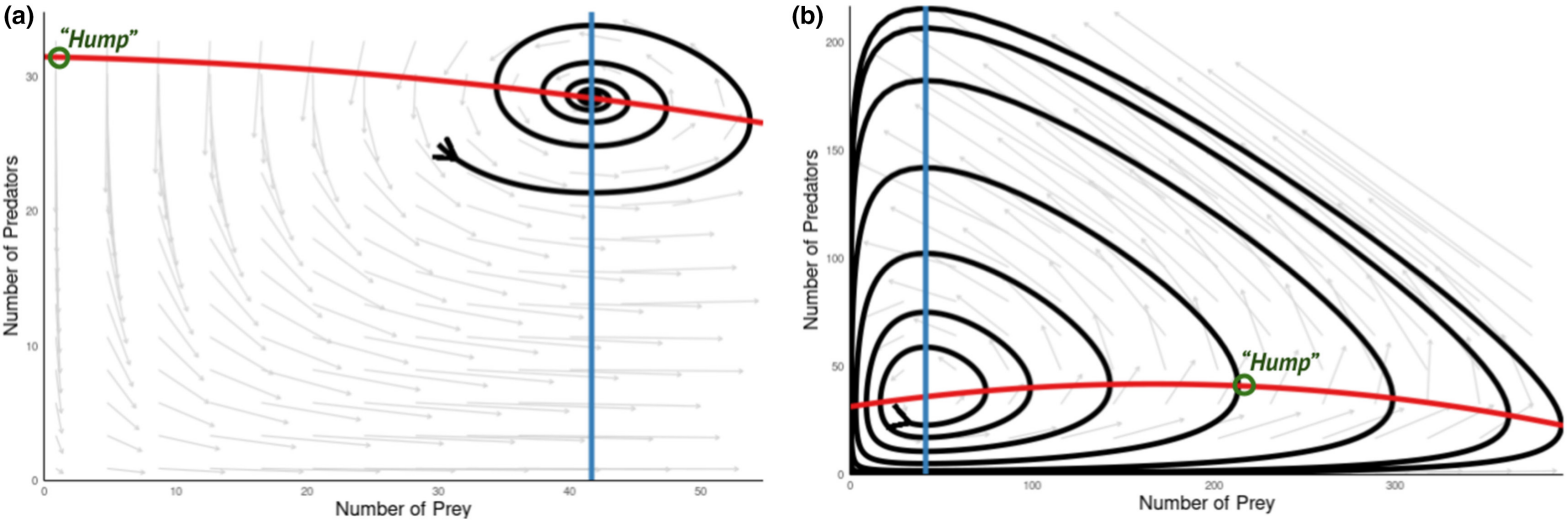
Screenshots of the predator–prey dynamics app being used to simulate the Rosenzweig‐MacArthur model. Panel a shows damped oscillations arising at low prey carrying capacities (*K* = 200) when the predator (blue) isocline intersects the prey (red) isocline to the right of the “hump”. In contrast, panel B shows the unstable oscillations that arise under high prey carrying capacity (*K* = 500) when predator isocline intersects the prey isocline to the left of the hump. Note that points and text indicating the location of the hump in prey isocline were added onto the screenshots.

Beyond the classroom, practicing theoreticians and empiricists alike can use Shiny apps to help communicate and learn insights from mathematical models. As a classic example, the paradox of enrichment (Rosenzweig, 1971) can be visualized with the predator–prey model app by altering the value of the prey carrying capacity (*K*) in the Rosenzweig‐MacArthur model tab. Low values of prey carrying capacity result in a stationary equilibrium or one with stable oscillations, while high values of prey carrying capacity — as might occur when a system is “enriched” — result in unstable oscillations that ultimately limit the system's persistence. In particular, careful exploration of the parameter *K* can reveal the logic behind Rosenzweig (1971)'s conclusion that the system can persist with stable oscillations or a stationary equilibrium only when the equilibrium point (intersection of the two isoclines) occurs to the right of the hump in the prey isocline (Figure 4).

Given the potential for interactive apps to substantially improve non‐theoreticians' interest in and understanding of mathematical models (see Figures 2 and 3), we echo Ou et al. (2022)'s suggestion that authors who publish new theoretical results can increase their reach by coupling manuscripts with interactive apps. A good example is the interactive exploration of Saavedra et al. (2017)'s results by Petry and Lepori (Petry & Lepori, 2022, https://ecodynamics.shinyapps.io/StructuralCoexistence/). Curating a centralized list of such apps that pair with newly published theoretical results is also one of the features we aim to add to the EcoEvoApps website.

### 3.3 Conclusions and outlook

Integrating theoretical and empirical approaches is often heralded as an ideal path for progress in ecology and evolutionary biology (Jeltsch et al., 2013; Laubmeier et al., 2020; Servedio, 2020; Shou et al., 2015), but such integration remains relatively limited (Scheiner, 2013). One likely barrier is that students are often not exposed to extensive quantitative training in traditional biology curricula (Chiel et al., 2010), and as a result, theoretical models remain intimidating for many empirical researchers (Grainger et al., 2022; Haller, 2014). While simulation‐based learning may not provide all the same insights as analytical solutions, platforms like R and Shiny allow us to build tools that give everyone easier access to theoretical insights that can otherwise take years of quantitative training to grasp. We leveraged these advances to build EcoEvoApps, a collection of interactive apps that allow users to interactively explore theoretical models, adding to a variety of existing interactive EEB education web resources such as Evo‐Ed (http://www.evo‐ed.org), HHMI BioInteractive (https://www.biointeractive.org), and Populus (Alstad, 2001). The key distinguishing features are that unlike these other resources, ecoevoapps is entirely open‐source, written in R, and allows readers to engage with models in multiple languages. As such, it is an equitable learning tool that is easily accessible and customizable by others in the global EEB community, where R is among the most commonly used programming languages (Gentleman et al., 2004; Lai et al., 2019). We see EcoEvoApps as a living project and a community resource. Moving forward, our priorities are to incorporate mathematical models from evolutionary biology and population genetics into the package to complement the current ecological focus, and to build on our preliminary evidence that interactive apps are useful tools for teaching quantitative models in classroom settings. We invite new collaborators who share our vision to contribute to the project.

## AUTHOR CONTRIBUTIONS

**Rosa McGuire:** Investigation (equal); methodology (equal); software (equal); validation (equal); visualization (supporting); writing – original draft (lead); writing – review and editing (equal). **Kenji Hayashi:** Methodology (equal); software (equal); writing – original draft (lead); writing – review and editing (equal). **Xinyi Yan:** Software (equal); validation (equal); writing – original draft (lead); writing – review and editing (equal). **Marcel Caritá Vaz:** Conceptualization (equal); investigation (equal); methodology (equal); software (equal); writing – original draft (supporting); writing – review and editing (supporting). **Damla Cinoğlu:** Software (supporting); writing – original draft (supporting); writing – review and editing (supporting). **Madeline Cowen:** Conceptualization (equal); software (equal); writing – original draft (supporting); writing – review and editing (supporting). **Alejandra Martínez‐Blancas:** Software (supporting); writing – original draft (supporting); writing – review and editing (supporting). **Lauren Sullivan:** Investigation (equal); methodology (equal); writing – review and editing (supporting). **Sheila Vazquez‐Morales:** Software (supporting); writing – original draft (supporting); writing – review and editing (supporting). **Gaurav Kandlikar:** Conceptualization (equal); formal analysis (lead); methodology (supporting); project administration (lead); resources (lead); software (lead); supervision (lead); visualization (equal); writing – original draft (equal); writing – review and editing (lead).

## Supporting information


Appendix S1‐S3

## Data Availability

The package code is freely available at https://gitlab.com/ecoevoapps/ecoevoapps/‐/releases/v1.1.0.resubmission. All data and code for classroom survey analyses are archived on Zenodo at https://doi.org/10.5281/zenodo.7332967.
